# Non-invasive detection of fasting blood glucose level via electrochemical measurement of saliva

**DOI:** 10.1186/s40064-016-2339-6

**Published:** 2016-05-23

**Authors:** Sarul Malik, Rajesh Khadgawat, Sneh Anand, Shalini Gupta

**Affiliations:** Center for Biomedical Engineering, Indian Institute of Technology (IIT) Delhi, New Delhi, 110016 Delhi India; Department of Endocrinology and Metabolism, All India Institute of Medical Sciences (AIIMS), New Delhi, 110016 Delhi India; Department of Biomedical Engineering, All India Institute of Medical Sciences (AIIMS), New Delhi, 110016 Delhi India; Department of Chemical Engineering, Indian Institute of Technology (IIT) Delhi, New Delhi, 110016 Delhi India

**Keywords:** Diabetes, Machine learning, Logistic regression, Artificial neural network, Support vector machine, Saliva

## Abstract

Machine learning techniques such as logistic regression (LR), support vector machine (SVM) and artificial neural network (ANN) were used to detect fasting blood glucose levels (FBGL) in a mixed population of healthy and diseased individuals in an Indian population. The occurrence of elevated FBGL was estimated in a non-invasive manner from the status of an individual’s salivary electrochemical parameters such as pH, redox potential, conductivity and concentration of sodium, potassium and calcium ions. The samples were obtained from 175 randomly selected volunteers comprising half healthy and half diabetic patients. The models were trained using 70 % of the total data, and tested upon the remaining set. For each algorithm, data points were cross-validated by randomly shuffling them three times prior to implementing the model. The performance of the machine learning technique was reported in terms of four statistically significant parameters—accuracy, precision, sensitivity and F1 score. SVM using RBF kernel showed the best performance for classifying high FBGLs with approximately 85 % accuracy, 84 % precision, 85 % sensitivity and 85 % F_1_ score. This study has been approved by the ethical committee of All India Institute of Medical Sciences, New Delhi, India with the reference number: IEC/NP-278/01-08-2014, RP-29/2014.

**Electronic supplementary material:**

The online version of this article (doi:10.1186/s40064-016-2339-6) contains supplementary material, which is available to authorized users.

## Background

Diabetes mellitus or hypoglycemia is a metabolic disorder that is characterized by high FBGL over a prolonged period of time. It is caused mainly due to two reasons—(1) insufficient production of insulin by the pancreas due to autoimmune destruction of the beta cells (Type-I) or, (2) sluggish response of the body cells to the insulin production by the pancreatic beta cells (Type-II) (Diabetes Mellitus 2005). In both cases, the produced insulin is either not enough for the body’s requirement or the body’s system becomes resistant to insulin. Gestational diabetes is another class of diabetes which is seen during pregnancy. During pregnancy the body becomes unresponsive towards insulin secretion due to the presence of human placental lactogen (Kim et al. 2002). The classical symptoms of diabetes include frequent urination, constant hunger and excessive thirst. Prolonged suffering from diabetes can lead to serious health conditions such as neuropathy, nephropathy, blindness, slow wound healing and many skin related complications (Diabetes Mellitus 2005).

The high rate of growth of diabetes, especially the Type-II kind, is attributed to obesity, poor nutrition, and lack of exercise in addition to genetic and environmental factors. A 2014 report by International Diabetes Federation (IDF) states that a whopping 387 million people suffer from diabetes worldwide (International Diabetes Federation 2014). The prevalence rate for diabetes is approximately 8.3 % out of which 46.3 % cases remain undiagnosed. Of these, almost 77 % of the diabetic cases are reported from low and middle income countries. Currently 62 million cases have been diagnosed with diabetes which is wining it the status of a potential epidemic in India. By 2035, India is predicted to become the diabetic capital of the world. Globally, diabetes cases are expected to increase to 592 million in the next two decades which is approximately double of the existing count.

One of the main objectives of our research was to develop an easy to use non-invasive method to classify FBGL, one of the prime indicators of diabetes, as high (≥120 mg/dl) or low (<120 mg/dl) in order to improve the overall experience of blood glucose measurement and as a result, quality of human life. BGL is currently determined using commercially available glucometers based on electrochemical changes in a drop of finger-pricked blood upon an enzymatic reaction on a test strip. This approach, although convenient and truly point-of-care, is invasive in nature as it poses risk for contamination and infection during the blood pricking process (Solnica et al. 2003). To address this issue, many researchers have explored other biological fluids such as saliva, urine, sweat or tears, that also contain residual amounts of glucose (Park et al. 2005; Srinivasan et al. 2003). Even intervening tissues such as bone or skin have been exploited for detection of trace amounts of glucose, however, their success has been limited due to practical limitations and weaker signals (Arnold and Small 2005; Rohrscheib et al. 2003). These efforts have led to many novel and innovative platforms for easy and continuous monitoring of BGL (Caduff et al. 2003; Cameron et al. 2000; Ellis and Goodacre 2006; Kost et al. 2000; Potts et al. 2002; Zarkogianni et al. 2015a; b).Glucose sensors have also been developed to evaluate glucose from tears by creating microelectrodes on polymer substrates shaped into a contact lens (Farandos et al. 2015; Liu 2015). Similarly, sweat has been used for the assessment of diabetes (Srinivasan et al. 2003).

Saliva is a complex biological fluid containing a cocktail of various hormones, antibodies, enzymes, growth factors and antimicrobial constituents (Lee and Wong 2009). In fact, most compounds found in the saliva are also present in the blood which make it functionally very similar to serum in reflecting the physiological state of the body (Schenkels et al. 1995). Monitoring of markers in saliva instead of serum is advantageous because saliva collection is a more straight forward and inexpensive process posing no risk of infection or discomfort to the patient (Lee and Wong 2009). It has been shown that diabetes mellitus affects the saliva composition, flow rate, buffering capacity, viscosity, electrolytic ionic composition and protein content quite significantly (Arana et al. 2006; Dodds and Dodds 1997; Dodds et al. 2000; Mata et al. 2004; Prathibha et al. 2013; Shirzaiy et al. 2013). Therefore, saliva is a well established biofluid for classifying individuals into diabetics and non-diabetics. Most of the diabetes-related studies involving saliva are however, based on protein analysis or specific biomarker measurement (Rao et al. 2009). No models exist for correlating or predicting FBGL from single or collective values of electrochemical variations in saliva as we demonstrate in our approach.

**Fig. 1:**
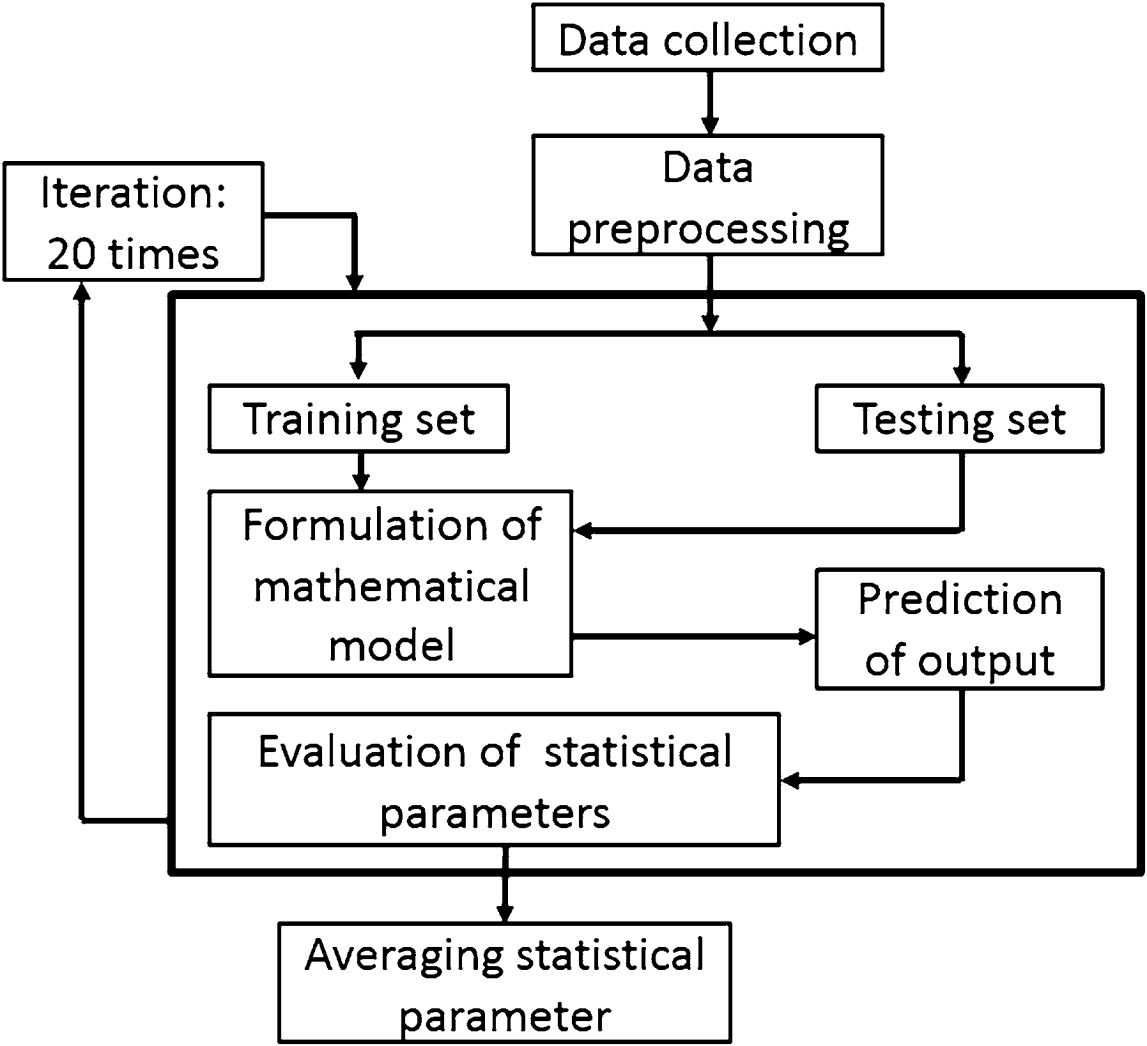
Algorithm applied for the detection of FBGLusing salivary electrochemical parameters

In this study, we carried out a detailed investigation of the electrochemical variations in saliva, collected from healthy and diabetic individuals, using well established machine learning algorithms. Parameters such as pH, oxidation redox potential (ORP), conductivity and individual concentration of sodium, potassium and calcium were statistically mapped against corresponding FBGL values determined under identical conditions (see process algorithm in Fig. 1). In addition to the electrochemical parameters, age was also taken as one of the key variables considering it is an important risk factor in manifestation of type 2 diabetes mellitus and cardiovascular diseases (Suastika et al. 2012). Three different mathematical models based on linear logistic regression (Peng et al. 2002), SVM (Cristianini and Shawe-Taylor 2000) and ANN (Sivanandam and Paulraj 2009) were applied to test which gave the best correlation for use of saliva as a facile biofluid for predicting FBGL. Logistic regression was used for its simplicity to estimate results in terms of end probabilities that lie in the range of 0 and 1 (Tabaei and Herman 2002). ANN was used because of its power to deal with ambiguous datasets and for performing pattern classifications (Principe et al. 1999). SVM was implemented as a potent algorithm to model highly complex and noisy data by transforming them from 2-D to multidimensional plane for better classification (Meyer and Wien 2015). The details and findings of our study are presented below.

## Methods

### Selection and organization of study groups

A total of 175 volunteers in the age range of 18–69 years were recruited for this study. The volunteers were divided into 2 groups—(1) Healthy volunteers (FBGL: 80–120 mg/dl; 41 female; 46 male; age range 18–62 years; mean age 35 ± 11 years), (2) Clinically diagnosed type II Diabetes Mellitus patients (FBGL ≥ 120 mg/dl; 47 female; 41 male; age range 21–69 years; mean age 47 ± 10 years). The following subjects were excluded from this study: (1) individuals with any salivary pathological condition such as salivary calculi, viral parotitis, (2) pregnant women, (3) people with gum bleeding, gingivitis or oral disorders such as oral cancer, (4) individuals with any other systemic sickness other than diabetes or severe diabetic complications, and (5) subjects on drugs like anticholinergic, sympathomimetic, skeletal muscle relaxant, antimigraine, cytotoxic, retinoids, anti HIV and cytokines which are known to affect the salivary flow rate and its composition. The inclusion criteria for a person suffering with diabetes mellitus was based on the recommendations of the Expert Committee on Diagnosis and Classification of Diabetes Mellitus (Kahn 2003). This included features of polydypsia, polyphagia, polyuria and elevated BGLs.

### Sample collection and analysis protocol

The participants were instructed to come in a fasting mode between 8:00 and 10:00 A.M. without brushing their teeth. They were then asked to swallow their existing saliva and made to sit on a comfortable chair in an isolated room keeping all ambient conditions the same so as to maintain their circadian rhythm. Every individual was asked to spit approximately 2 mL of saliva in a pre-autoclaved collecting vial. These saliva samples were then immediately analyzed for various electrochemical parameters before they could degrade proteolytically. The pH and oxidation reduction potential (ORP) values were measured using the F-71 Laqua Lab (Japan) pH/ORP meter. The conductivity and concentration of the electrolytes (mainly Na^+^, K^+^, and Ca^++^) were recorded using the Horiba Laqua twin series ion selective models (Malik et al. 2015). For comparison with the current gold standard, the FBGL of all the volunteers was also measured in the venous plasma and analyzed by an automatic biochemical analyzer (Cobas integra 400 plus).

### Data preprocessing

The electrochemical data obtained from the saliva samples were used to train machine learning algorithms such as logistic regression, SVM and ANN in order to be able to predict the results for unknown samples in future. Machine learning recognizes patterns and mining trends in large data sets and is now routinely used in pharmaceutical industry to meet their targets. In our study, the mathematical models were coded in MATLAB R2014a (version 8.3). Prior to data fitting, an essential feature scaling operation was performed on all the different parameters, namely pH, ORP, conductivity, electrolyte concentration and volunteer’s age, to obtain normalized data in the range of −1 to 1. This was done to avoid any bias generated by the differences in the parameter measuring units. The relationship used for feature normalization is shown in Eq. 1,

1 $$x_{i}^{{\prime }} = \frac{{x_{i} - \mu }}{\sigma }$$

where, $x_{i}$ is the input feature variable (pH, ORP, age etc.), $x_{i}^{{\prime }}$ is the normalized feature variable, and $\mu$ and $\sigma$ are the mean and standard deviations from all the data obtained for that feature. The FBGL values measured in the venous plasma were classified as 1 (high FBGL) if ≥120 mg/dl else 0, and fitted against the normalized training set data to determine the coefficients of the fitted variables related by the general equation (Eq. 2),

2 $$Y = f\_\theta \left( x \right).$$

Here, *Y* is the predicted output FBGL value of either 0 or 1, *x* represents either linear or non-linear combination of input variables and *θ* is the coefficient value corresponding to *x*.

Once the entire data from 175 volunteers were normalized, they were cross-validated three times by dividing into three equal randomly generated data sets. At a time, two random data sets were used for training and the third one was used for testing. Since the process was cross-validated three times, it generated three different combinations of training and testing set in one complete cycle. The motivation to do this was to create a shuffled training and testing data set with no biasing. The training set was then used to train the algorithm which in turn provided a model for FBGL prediction of 0 or 1. The testing set was used to evaluate the utility of the trained model by computing the average values of the reported data and the classifier performance index (CPI) parameters (discussed later) after twenty iterations of the algorithm. The entire process cycle was iterated 20 times by randomly selecting different combinations of cross-validated training and testing data sets to further enhance the fitting accuracy and give much more stable results. The final outcome was reported as an average result of the above discussed process.

### Logistic regression method

A linear logistic regression model was developed to detect high FBGL from age and salivary electrochemical parameters. The logistic regression model generates output in terms of probabilities and we chose 0.5 as the threshold equivalent to 120 mg/dl of BGL (Malik et al. 2015). Predicted output value (POV) depends on the input variables $x_{i }$ and their coefficients $\theta_{i}$ as shown in Eq. 3 below,

3 $$POV = \frac{1}{{1 + e^{{ - \left( {\theta_{0} + \sum_{1}^{n} \left( {x_{i} \theta_{i} } \right)} \right)}} }}$$

The values of $\theta_{i}$ were initialized to zero to keep the initial condition unbiased since the data was normalized and separated around zero. Then the gradient descent algorithm was applied to the training data set to calculate the values of the coefficients using the mean square error (MSE) method (Additional file 1).

### Artificial neural network (ANN)

ANN is another machine learning tool that can be used for fitting non-linear functions with higher precision and accuracy to analyze associated complex patterns (Chen and Billings 1992). We used a feed-forward ANN with back propagation gradient descent algorithm to classify the diabetic patients from normal ones using their salivary data. The ANN classifier architecture consisted of an input layer with 7 neurons (one for each parameter), 33 hidden layer neurons and two nodes in output layer with one neuron each (Additional file 1: Fig. S1). The 33 hidden layer neurons architecture was chosen as it gave us maximum accuracy with minimum deviations (see Additional file 1: Fig. S2). The ANN was trained by reducing the MSE of the training dataset (Additional file 1: Fig. S3). Once the MSE was minimized, the values of the constants obtained were stored internally to validate the model using half of the remaining data. The results of validation created a platform for testing the model by the other half of the remaining data (Additional file 1: Fig. S3)

### Support vector machine (SVM)

SVM is another powerful tool now routinely used in clinical applications (Cortes and Vapnik 1995; Maglogiannis et al. 2009). In our study, it was used to map the salivary data from a lower to multidimensional feature space such that the high and low FBGL could be separated with maximum margin by a hyperplane using various non-linear kernels as shown in Eq. 4 (Cristianini and Shawe-Taylor 2000). Here, $x_{i}$ is the normalized feature vector and $x_{j}$ is the support vector.

4 $$k\left( {x_{i} ;x_{j} } \right) = f\left( {x_{i} } \right)^{T} f\left( {x_{j} } \right)$$

5 $$k_{linear} \left( {x_{i} ;x_{j} } \right) = x_{i}^{T} x_{j}$$

6 $$k_{Gaussian} \left( {x_{i} ;x_{j} } \right) = exp^{{ - \gamma \parallel x_{i} - x_{j} \parallel^{2} }} .$$

The SVM classifier was implemented using the LibSVM software package in MATLAB (Chang and Lin 2011) using the linear and Gaussian (radial basis function; RBF) kernel functions represented in Eqs. 5 and 6, respectively (Thurston et al. 2009). To develop an optimal SVM model, two key parameters, *C* and γ, were preselected for the kernels. *C* is commonly known as the penalty parameter which controls over-fitting of the model. In case of RBF, the classification is generally better due to a higher value of *C* which makes the SVM classify more correctly. Parameter γ controls the degree of non-linearity of the model. *C* is commonly used in implementing linear as well as RBF, whereas γ is used specifically for the RBF kernel (see Additional file 1: Fig. S5).

### Classifier performance index (CPI)

**Fig. 2:**
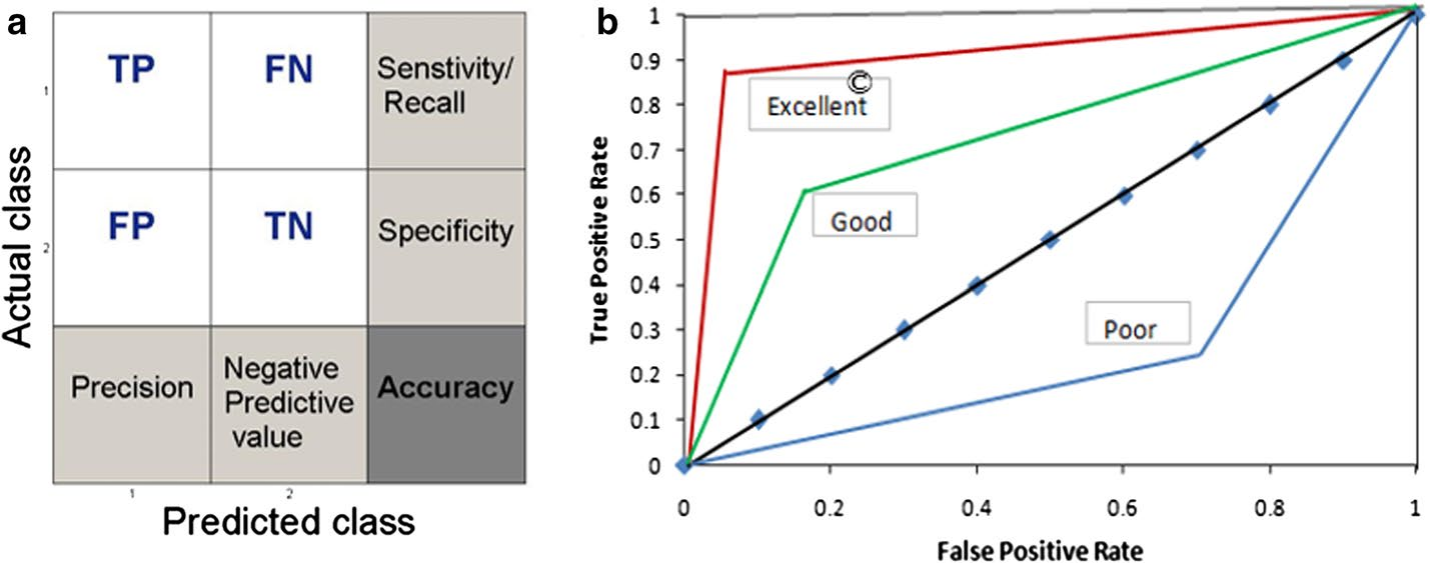
**a** Layout of the confusion matrix showing various statistical performance indices used for validating our model fitting process. **b** General description of the ROC performance

The model performances were determined using the confusion matrix (also known as error or contingency matrix in machine learning) and the receiver operating characteristic (ROC) curve (Qin 2005) (Fig. 2a). True positives (TP) were defined as the cases where both the actual and predicted values of the FBGL lied in the ≥120 mg/dl range. Similarly, true negatives (TN) were cases where both the actual and predicted values had FBGL <120 mg/dl. False positives (FP) represented cases where the actual state of disease was false but the model predicted them to be true, and vice versa for false negatives (FN). The data in the confusion matrix were used to estimate a set of statistically-relevant performance indicators defined below,

7 $$Accuracy = \frac{TP + TN}{TP + TN + FP + FN},$$

8 $$Presicion = \frac{TP}{TP + FP},$$

9 $$Recall = \frac{TP}{TP + FN},$$

10 $$F_{1} \;score = \frac{2TP}{2TP + FP + FN}.$$

Accuracy provides the total count of correctly predicted high FBGL cases by the model in a total participating population (Eq. 7). The precision gives the fraction of actual to detected diseased cases (Eq. 8). Similarly, recall or sensitivity gives an estimate of the truly detected diseased cases among the actual ones (Eq. 9). F_1_ score is another important parameter defined as the harmonic mean of recall and precision (Eq. 10). It is used when we want to contrast the performances of different prediction parameters with a single evaluation matrix to judge the classifier efficiency. For definitions of other CPIs such as specificity and negative predictive value, see Additional file 1.

In the ROC curve, a well-accepted graphical tool for performance illustration of a binary classifier (Slaby 2007), the True Positive Rate (TPR) is plotted on the y-axis against the False Positive Rate (FPR) on the x-axis TPR is mathematically the same as recall (Eq. 11), whereas, FPR (Eq. 12) signifies how many wrong positive results occur among all the negative samples available during the test. To obtain a reasonable performance of the binary classifier, the ratio of TPR to FPR should be high.

11 $$True\;positive\;rate = \frac{TP}{TP + FN}$$

12 $$False\;positive\;rate = \frac{FP}{FP + TN}$$

## Results and discussion

A mathematical model is used to quantify the effect of different parameters on a system and to predict its behavior. Feature normalization or scaling of the data is often the first step required to nullify the effect of different measurement units. Finally, cross-validation helps avoid over-fitting during performance evaluation of a classifier. Below we discuss our findings obtained from fitting the three different mathematical models to our volunteer test data.

### Logistic regression results

**Fig. 3:**
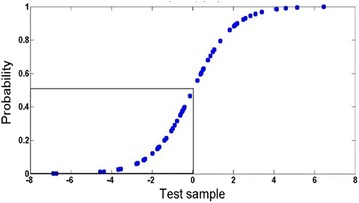
A sigmoidal probability distribution curve obtained by logistic regression fitting of the test data

**Fig. 4:**
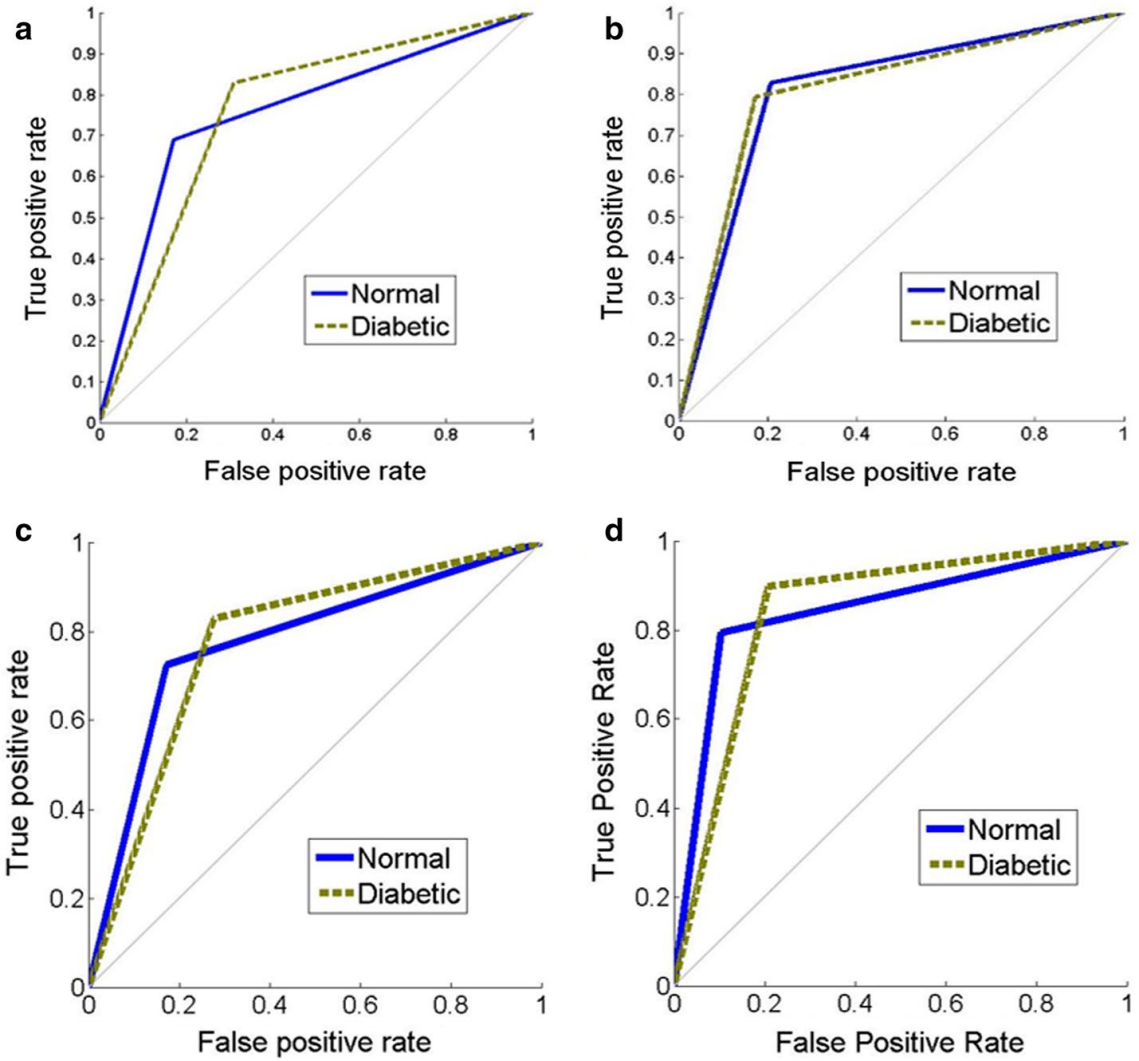
**a** The ROC plots for linear logistic regression model. The coordinates for the normal and diabetic populations were (0.69, 0.16) and (0.82, 0.31), respectively. **b** The ROC plots for the ANN model illustrating the coordinates to be closely placed at (0.84, 0.2) for the normal class and at (0.8, 0.16) for the diabetic population. **c** The ROC plots for linear-SVM. **d** The ROC plots for RBF-SVM models. The coordinates for the curves were (0.72, 0.16) for the normal class and (0.84, 0.28) for the diabetic class in linear, and (0.8, 0.1) for the normal class and (0.9, 0.2) for the diabetic class in RBF-SVM. The RBF-SVM ROC coordinates being closer to (1, 0) suggested a better fit than the linear model

The probability distribution curve drawn for a randomly selected set of test data exhibited a sigmoidal behaviour as expected (Fig. 3). A threshold value of 0.5 (equivalent to 120 mg/dl FBGL) on this curve was chosen to classify an individual with high FBGL. The efficiency of our logistic regression algorithm when evaluated using the confusion matrix gave average CPI values in the range of 75–77 % (Table 1). The coordinates of ROC plots corresponding to the performance of the algorithm were seen to be (0.69, 0.16) for the healthy class with low FBGL and (0.82, 0.31) for the diabetic patients with high FBGL (Fig. 4a). These results implied that the overall ability of the model to discriminate between high and low FBGL was not as high as desired. The low efficiency of the algorithm is likely due to the intrinsic nature of the logistic regression model used, which assumes that the output is some linear function of the input variables. To increase the model performance efficiency that is suitable for clinical accuracy, one can use non-linear models that can handle even ambiguous data. The only challenge, however, is that the data handling and interpretation complexity increases drastically with the number of multiple variable combinations. There are well known non-linear supervised learning algorithms like ANN and SVM available that can deal with the intricacies of automatic and random data set generation, training and validation. Therefore, we next investigated both these model approaches to see which one gave us higher performance efficiency.

**Table 1:** Final output of the CPI parameters obtained after twenty iterations of linear logistic regression, ANN, linear- and RBF-SVM models

S. no.	Machine learning technique	Computational parameters			
		Accuracy	Precision	Recall	F_1_ score
1	Linear logistic regression	75.86 ± 2.3	76.76 ± 3.8	75.48 ± 5.4	75.71 ± 2.6
2	ANN	80.7 ± 2.1	81.2 ± 1.7	79.3 ± 3.4	80.2 ± 2.2
3	Linear-SVM	77.93 ± 2.7	77.59 ± 3.5	79.43 ± 4.7	78.11 ± 2.7
4	RBF-SVM	84.09 ± 2.8	83.75 ± 3.3	84.92 ± 4.5	84.06 ± 2.9

### ANN results

ANN is a supervised learning technique that has shown excellent performance not only in pattern recognition but also in various classification problems. The best performance achieved after system validation was at the cross-entropy value of 0.44 obtained after 10 iterations (Additional file 1: Fig. S3). The statistical significance of the model’s competence was verified by determining that the training, validation and testing data followed a normal distribution curve (Additional file 1: Fig. S4). The CPIs for the ANN model (Table 1) and the corresponding ROC (Fig. 4b) indicated that the fitted values improved in comparison to the logistic regression model. To further increase the prediction accuracy, we next tried the SVM algorithm on our experimental data.

### SVM results

In SVM model fitting, the values of *C* and γ were first optimized to attain maximum accuracy of the RBF kernel (Additional file 1: Fig. S5). Next, the CPIs were calculated for both the linear and Gaussian SVM algorithms (Table 1). The RBF-SVM model was able to classify high and low FBGL not only with higher accuracy than before but also with greater sensitivity (or, recall) as compared to the earlier models. The linear-SVM values, however, stayed more or less the same as before indicating that the data had some intrinsic non-linear behavior. The ROC plots for linear and Gaussian kernels again gave similar results illustrating that the RBF-SVM is better suited for detecting volunteers with high FBGL (Fig. 4c, d).

On comparing between all the three models, RBF-SVM gave the highest accuracy of approximately 85 % for classifying TP and TN population among all the volunteers. Similarly, the other CPI values for RBF-SVM were also on average higher than the other two algorithms (Additional file 1: Fig. S6). One-way analysis of variance (ANOVA) and paired t test both confirmed the statistically significant higher performance of the RBF-SVM model (Additional file 1: Table S1). Maximum deviation was seen in recall values, whereas the variability in the rest of the three parameters was not as high. Considering that the linear logistic regression gave the poorest fit out of all the three classifiers, we believe the data to be correlated in a highly non-linear fashion to each other. Although logistic regression is a potent classifier for numerous applications, it was unable to detect high FBGL using salivary electrochemical parameters. In future, other experimental parameters of saliva such as anion concentration etc. or a characterisitic of an individual like sex, body mass index, etc. may also be included in the modeling algorithm to increase the accuracy of our approach.

## Conclusions and perspectives

We applied known machine learning techniques to demonstrate the potential use of saliva as an alternate biofluid (other than blood) to predict FBGL in healthy and diabetic patients. In addition, using the RBF-SVM model, we could detect the FBGL values to lie either above or below 120 mg/dl with approximately 85 % accuracy. This accuracy level is based on highly stringent conditions of zero error but considering that the 2014 FDA guidelines allow the commercial blood glucometers to operate with high standard deviations of greater than 15 % (±15 mg/dL for BGL < 75 mg/dL and ± 20 mg/dL > 75 mg/dL), our results show significant correlation with the actual BGL values. In future, the accuracy of our technique may be further improved by including more statistically relevant parameters (body mass index etc.) and by increasing the number of subjects in the database. Eventually, using latest principles of microfabrication, multiple commercial ion-selective sensors could be miniaturized into a single integrated electrochemical measurement device for point of care usage. This would not only help overcome the present day challenges of measuring BGL, which can be as many as eight times a day in case of admitted patients, saving patients a lot of discomfort but also greatly improve the quality of healthcare by providing a risk-free method for BGL monitoring without fear of secondary contamination. Finally, we strongly believe that the electrochemical variations in saliva could have a huge potential for detection of FBGL.

## Additional file

10.1186/s40064-016-2339-6 Supplementary information.
