# Supplementary material for: Non-invasive detection of fasting blood glucose level via electrochemical measurement of saliva
Source: Springerplus. 2016 May 23;5(1):701. doi: 10.1186/s40064-016-2339-6 (PMC4899397; doi:10.1186/s40064-016-2339-6)
Supplement: Supplementary file 1 — 10.1186/s40064-016-2339-6 Supplementary information. [file 40064_2016_2339_MOESM1_ESM.docx]

Non–Invasive detection of fasting blood glucose level via electrochemical measurement of saliva

Sarul Malik^1^, Rajesh Khadgawat^2^, Sneh Anand^1,3^ and Shalini Gupta^4^

*^1^ Center for Biomedical Engineering, Indian Institute of Technology (IIT) Delhi, Hauz Khas, India 110016*

*^2^Dept. of Endocrinology and Metabolism, All India Institute of Medical Sciences (AIIMS), Delhi, India 110016*

*^3^Dept. of Biomedical Engineering, AIIMS, India 110016*

*^4^Dept. of Chemical Engineering, IIT Delhi, Hauz Khas, India 110016*

*Corresponding author: shalinig@chemical.iitd.ac.in*

**II. Methodology**

## **Logistic regression**

Mean squared error (MSE): It is defined as the averaged measure of the square of the errors. The error is the measure of the difference between the predicted value $h_{\theta}\left( x^{i} \right)$and actual value $y^{i}$. The MSE is represented by $J\left( \theta\right)$ for $m$ number of observations in eqn. 1

$J\left( \theta\right)=\frac{\sum_{i=1}^{m}{(h_{\theta}\left( x^{i} \right)-y^{i})}^{2}}{m}$ (1)

## **Artificial neural network (ANN)**

The ANN classifier architecture used to classify the high FBGL consisted of an input layer with 7 neurons (one for each parameter), 33 hidden layer neurons and two nodes in output layer with one neuron each. Fig. S1. is the schematic of the artificial neural network’s architecture for different salivary electrochemical parameters and volunteer’s age as input.


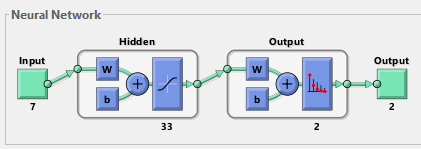


Fig. S1. A schematic of the ANN layout, with 7 input, 33 hidden layer neurons and 2 output layers, used in our study.


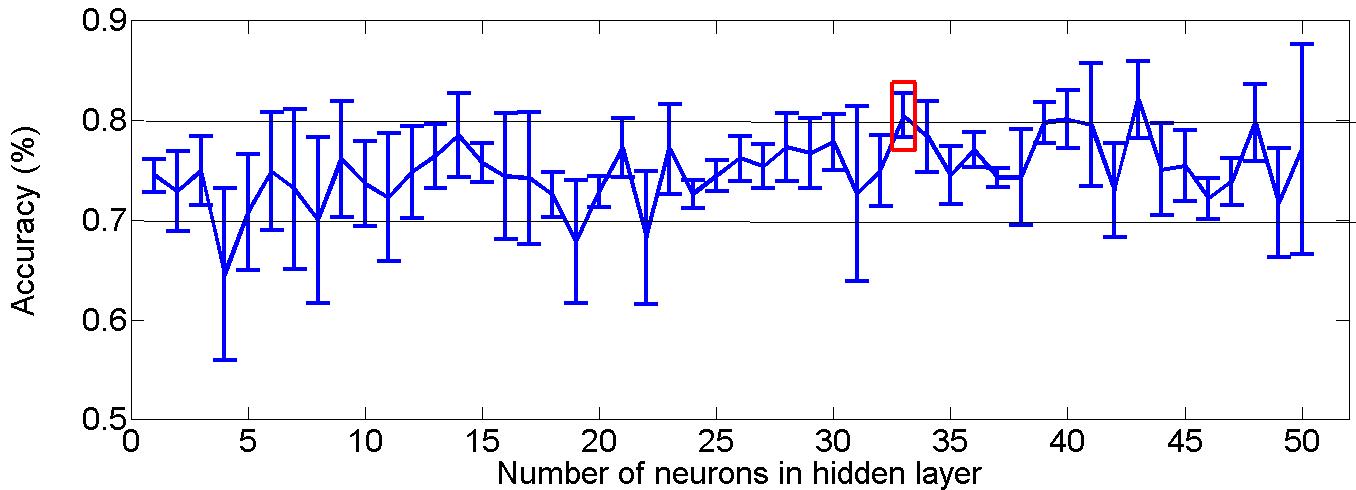


Fig. S2. The variations in accuracy corresponding to hidden layer neurons.

Fig. S2 represents the reason for choosing 33 hidden layer neurons in this study. The maximum accuracy came out to be 82% with 43 hidden layer neurons but we chose 33 hidden layer neurons which could predict output with an accuracy of 80%. This was because the standard deviation associated with the 43 neurons was more as compared to 33 neurons.

The performance of the neural network could be measured in terms of the MSE which is shown on the log scale in Fig. S3. The MSE should rapidly decrease as the network gets trained using training set. The performance of training, validation and test set is also shown in the below figure which provides an estimate of MSE in response to the validation of the network. The best validation performance was 0.44 obtained at epoch 10 for this network. After validation of the neural network, the model parameters got stored internally and used by test data for prediction of the output.


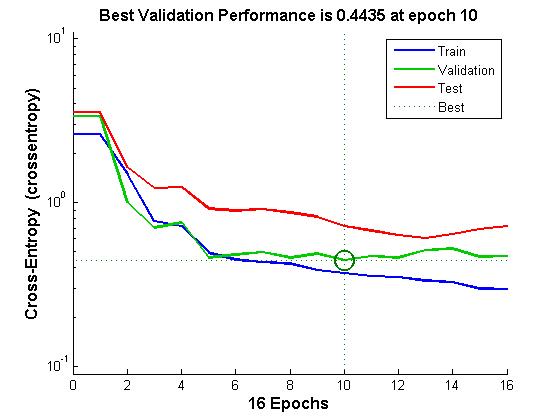


Fig. S3. Performance of the ANN in terms of error


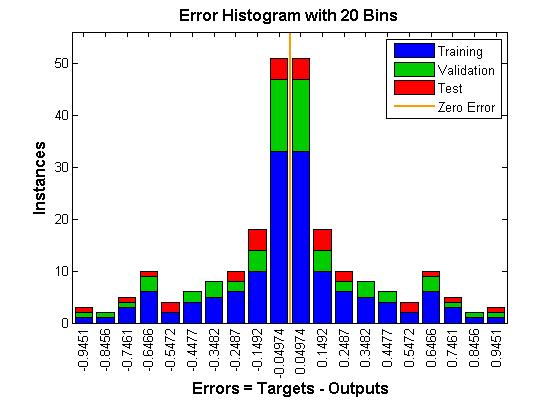


Fig. S4: Error histogram corresponding to the performance of ANN

## Support vector machine

Fig. S5. was obtained by constructing the heat map for the salivary electrochemical parameters and volunteer’s age as inputs to predict their FBGL using RBF kernel. As defined in the manuscript, “C” is known as the penalty parameter which controls the over-fitting of the model and parameter “γ” controls the non-linearity of the model. To optimize the value of C and γ, this heat map was drawn in order to obtain the best value of CPIs. The maximum value of accuracy was obtained as ~ 85% at C = 100000 and γ = 0.01. The different values for accuracy could be seen for different C and γ as a measure of the color approaching from blue to brown.


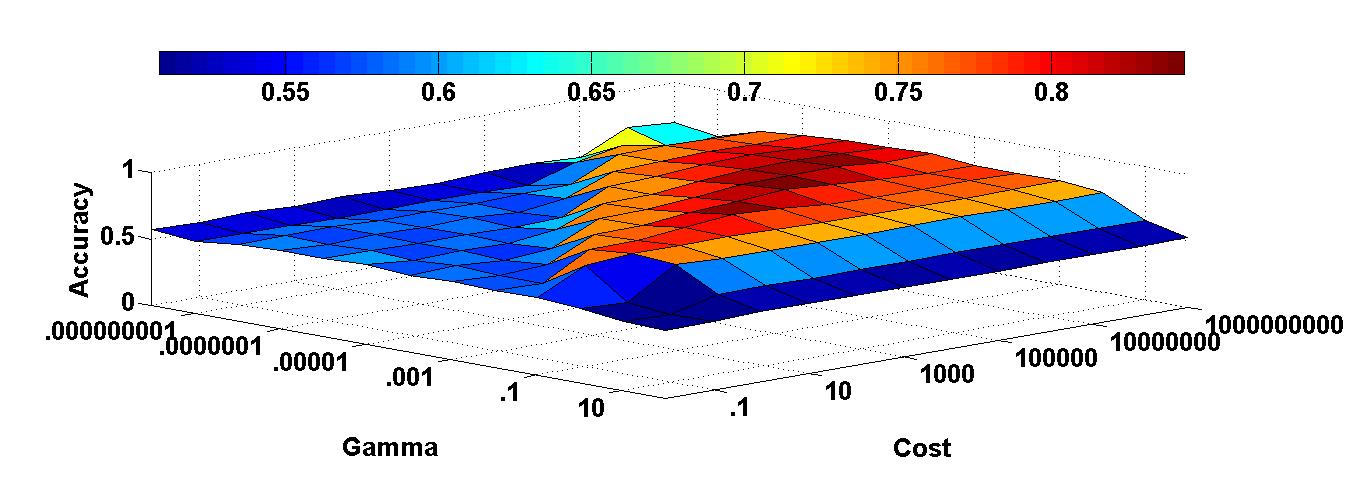


Fig. S5. Accuracy of the RBF kernel for different values of γ (left) and *C* (right).

## Classifier performance index (CPI)

Recall or sensitivity is another significant parameter which gives us an estimate of the truly predicted high FBGL among the actual ones(**eqn. S1**). Specificity and negative predictive value are two others sets of statistically significant parameters which are concerned with the false alarms of the classifier. Specificity represents the percentage of healthy population predicted correctly by the model (**eqn.S2**). Negatitive predicted value is the percentage of actual to the overall healthy cases predicted by the model (**eqn.S3**). It is similar in concept to the Precision parameter used for identifying high FBGL.

$Recall=\frac{TP}{TP+FN}$ (S1)

$Specificity=\frac{TN}{FP+TN}$ (S2)

$Negative predictive value=\frac{TN}{TN+FN}$ (S3)

# **Results and discussion**


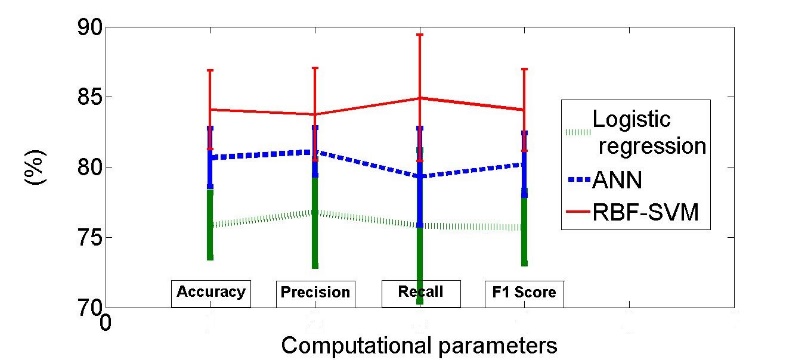


Fig. S6. Comparison of all the three machine learning techniques used for FBGL detection using saliva. RBF-SVM showed the best fitting.

Table S1. P-values obtained by applying ANOVA and t-test to compare the models’ predictive performance in terms of CPIs. The highlighted values indicate levels where P value obtained was > 0.05.

| Computational parameter | Algorithm (i) | Algorithm (j) | P value (ANOVA) | P value (paired t-test) |
| --- | --- | --- | --- | --- |
| Accuracy | LR | ANN | 0.000 | 0.000 |
|  |  | Linear-SVM | 0.195 | 0.030 |
|  |  | RBF-SVM | 0.000 | 0.000 |
|  | ANN | LR | 0.000 | 0.000 |
|  |  | Linear-SVM | 0.009 | 0.003 |
|  |  | RBF-SVM | 0.000 | 0.000 |
|  | Linear-SVM | LR | 0.195 | 0.030 |
|  |  | ANN | 0.009 | 0.003 |
|  |  | RBF-SVM | 0.000 | 0.000 |
|  | RBF-SVM | LR | 0.000 | 0.000 |
|  |  | ANN | 0.000 | 0.000 |
|  |  | Linear-SVM | 0.000 | 0.000 |
| Precision | LR | ANN | 0.029 | 0.006 |
|  |  | Linear-SVM | 0.999 | 0.887 |
|  |  | RBF-SVM | 0.000 | 0.000 |
|  | ANN | LR | 0.029 | 0.006 |
|  |  | Linear-SVM | 0.040 | 0.006 |
|  |  | RBF-SVM | 0.000 | 0.001 |
|  | Linear-SVM | LR | 0.999 | 0.887 |
|  |  | ANN | 0.040 | 0.006 |
|  |  | RBF-SVM | 0.000 | 0.000 |
|  | RBF-SVM | LR | 0.000 | 0.000 |
|  |  | ANN | 0.000 | 0.001 |
|  |  | Linear-SVM | 0.000 | 0.000 |
| Recall | LR | ANN | 0.000 | 0.000 |
|  |  | Linear-SVM | 0.008 | 0.005 |
|  |  | RBF-SVM | 0.000 | 0.000 |
|  | ANN | LR | 0.000 | 0.000 |
|  |  | Linear-SVM | 0.726 | 0.229 |
|  |  | RBF-SVM | 0.000 | 0.000 |
|  | Linear-SVM | LR | 0.008 | 0.005 |
|  |  | ANN | 0.726 | 0.229 |
|  |  | RBF-SVM | 0.000 | 0.000 |
|  | RBF-SVM | LR | 0.000 | 0.000 |
|  |  | ANN | 0.000 | 0.000 |
|  |  | Linear-SVM | 0.000 | 0.000 |
| F1score | LR | ANN | 0.000 | 0.000 |
|  |  | Linear-SVM | 0.076 | 0.014 |
|  |  | RBF-SVM | 0.000 | 0.000 |
|  | ANN | LR | 0.000 | 0.000 |
|  |  | Linear-SVM | 0.030 | 0.008 |
|  |  | RBF-SVM | 0.000 | 0.000 |
|  | Linear-SVM | LR | 0.076 | 0.014 |
|  |  | ANN | 0.030 | 0.008 |
|  |  | RBF-SVM | 0.000 | 0.000 |
|  | RBF-SVM | LR | 0.000 | 0.000 |
|  |  | ANN | 0.000 | 0.000 |
|  |  | Linear-SVM | 0.000 | 0.000 |

In order to investigate whether there exist significant differences between the models’ predictive performances, the one-way analysis of variance (ANOVA) was applied using SPSS tool keeping the level of significance as 0.05. The ANOVA revealed statistically significant differences in accuracy (p value .000), precision (p value .000), recall (p value .000), and F1-score (p value .000) between RBF-SVM and other classifiers - logistic regression, ANN and linear-SVM (Table S1). All classifiers showed significant difference between their performance except logistic regression and linear-SVM classifier. In order to perform a pairwise comparison of the models’ accuracy, the paired t-test was applied and the cutoff of 0.05 was considered as the level of significance. In Table S1, the corresponding p values are presented for all the classifiers. It can be concluded that RBF-SVM is a potent classifier to detect FBGL from saliva.
